# Bringing the Bugs Back In: Environmental Health Research Model Combines Toxicology and Infectious Disease

**DOI:** 10.1289/ehp.118-a353b

**Published:** 2010-08

**Authors:** Harvey Black

**Affiliations:** **Harvey Black** of Madison, WI, has written for *EHP* since 1994. His work has also appeared in *Environmental Science & Technology*, *ChemMatters*, and the *Milwaukee Journal Sentinel*

Although pathogens are known to modify the effects of toxicants, U.S. environmental health research currently focuses on physical agents and chemical toxicants—a focus that limits the field by ignoring the interaction between pathogens and toxic agents **[*EHP*** **118(8):1165–1172; Feingold et al.]**. These authors present a conceptual paradigm that integrates infectious disease and toxicologic environmental health research, promotes cross-disciplinary education and communication, and elucidates a fuller body of environmental health risk factors.

Chemical toxicity often involves relatively direct effects of exposures on health outcomes, but infectious disease transmission typically is more complex, depending on factors such as dynamic environmental and ecologic systems, patterns of contact among populations, and host immune status. But interactions between pathogens and toxicants are undeniable. For instance, hepatitis B virus and aflatoxin individually increase the risk of liver cancer, but combined exposure to both agents increases risk far more than would be expected based on effects of the two risk factors in isolation. And in the case of cervical cancer, although infection with human papillomavirus is believed to be necessary for the cancer to occur, smoking may act as a cofactor and increase the risk the cancer will occur in someone infected with the virus.

The authors identify multiple points between initial exposure and clinical disease at which toxicant–pathogen interactions can occur. They also describe approaches common to both areas of research. Both focus on upstream interventions to prevent disease by preventing exposure. Both areas also focus on spatial context (i.e., proximity to toxic or pathogenic agents) and quantitative modeling to estimate exposure, and both use biomarkers to study exposure, susceptibility, and disease.

Fostering collaborations between researchers in these fields can lead to a better understanding of complex exposures and resulting diseases. “Classic reductionist thinking in toxicology focuses on ‘one toxicant, one outcome’ research,” the authors write. In contrast, they conclude, “If basic research is to increase our ability to predict the consequences of exposure to environmental chemicals, we must embrace nonreductionist thinking and design experimental models that emulate human experience.”

## Figures and Tables

**Figure:**
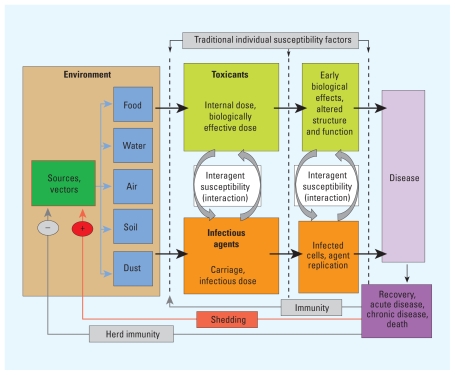
Pathogen–toxicant interactions may influence the progression from exposure to disease at multiple points.

